# NRSF and BDNF polymorphisms as biomarkers of cognitive dysfunction in adults with newly diagnosed epilepsy

**DOI:** 10.1016/j.yebeh.2015.11.013

**Published:** 2016-01

**Authors:** Alix Warburton, Fabio Miyajima, Kanvel Shazadi, Joanne Crossley, Michael R. Johnson, Anthony G. Marson, Gus A. Baker, John P. Quinn, Graeme J. Sills

**Affiliations:** a Department of Molecular and Clinical Pharmacology, Institute of Translational Medicine, University of Liverpool, Liverpool L69 3GL, UK; b Department of Medicine, Imperial College London, London SW7 2AZ, UK

**Keywords:** AED, antiepileptic drug, AMIPB, Adult Memory and Information Processing Battery, AVLT, Auditory Verbal Learning Task, β, beta coefficient, BDNF, brain-derived neurotrophic factor, CI, confidence interval, CVST, Computerized Visual Search Task, HWE, Hardy–Weinberg equilibrium, htSNP, haplotype-tagging SNP, LD, linkage disequilibrium, MAF, minor allele frequency, NRSF, neuron-restrictive silencer factor, SNP, single nucleotide polymorphism, REML, Restricted Maximum Likelihood, REST, restrictive element-1 silencing transcription factor, SANAD, Standard and New Antiepileptic Drug, VNTR, variable number tandem repeat, VRT, visual reaction time, BDNF, Cognition, Epilepsy, NRSF/REST, Biomarkers

## Abstract

Cognitive dysfunction is a common comorbidity in people with epilepsy, but its causes remain unclear. It may be related to the etiology of the disorder, the consequences of seizures, or the effects of antiepileptic drug treatment. Genetics may also play a contributory role. We investigated the influence of variants in the genes encoding neuron-restrictive silencer factor (NRSF) and brain-derived neurotrophic factor (BDNF), proteins previously associated with cognition and epilepsy, on cognitive function in people with newly diagnosed epilepsy. A total of 82 patients who had previously undergone detailed neuropsychological assessment were genotyped for single nucleotide polymorphisms (SNPs) across the NRSF and BDNF genes. Putatively functional SNPs were included in a genetic association analysis with specific cognitive domains, including memory, psychomotor speed, and information processing. Cross-sectional and longitudinal designs were used to explore genetic influences on baseline cognition at diagnosis and change from baseline over the first year since diagnosis, respectively. We found a statistically significant association between genotypic variation and memory function at both baseline (NRSF: rs1105434, rs2227902 and BDNF: rs1491850, rs2030324, rs11030094) and in our longitudinal analysis (NRSF: rs2227902 and BDNF: rs12273363). Psychomotor speed was also associated with genotype (NRSF rs3796529) in the longitudinal assessment. In line with our previous work on general cognitive function in the healthy aging population, we observed an additive interaction between risk alleles for the NRSF rs2227902 (G) and BDNF rs6265 (A) polymorphisms which was again consistent with a significantly greater decline in delayed recall over the first year since diagnosis. These findings support a role for the NRSF–BDNF pathway in the modulation of cognitive function in patients with newly diagnosed epilepsy.

## Highlights

• Cognitive dysfunction is a common and important comorbidity in people with epilepsy.
• Causes of cognitive problems are unknown, but genetics may be involved.
• We compared *NRSF* and *BDNF* gene variants with cognition in new-onset epilepsy.
• Deterioration in memory task scores from baseline was associated with genotype.
• The NRSF/BDNF pathway may be relevant to cognitive function in people with epilepsy.

## 1 Introduction

Cognitive decline is a common and important comorbidity in people with epilepsy and is well documented in those with long-standing intractable seizures [1], [2], [3], [4], [5]. In contrast, comparatively few studies have explored the natural history of cognitive function in cohorts of patients with newly diagnosed epilepsy [3], [4], [6], [7], [8]. One such study enrolled a subgroup of participants from the UK-based Standard and New Antiepileptic Drug (SANAD) trials [9], [10] and assessed their long-term cognitive outcomes using a neuropsychological test battery performed at initial presentation (baseline) and again at 12 months and an average of 5 years thereafter. Individuals with new-onset epilepsy were significantly impaired, in comparison to healthy controls, in cognitive domains specifically relating to memory, psychomotor speed, and information processing both at baseline, using a cross-sectional design, and over the first 5 years of treatment, using a longitudinal design [3], [4]. A number of studies support this finding, albeit with some inconsistencies in the cognitive domains affected [11], [12], [13], [14], [15], [16], [17], [18], but there are also contradictory reports in the literature that suggest improvements or at least no change in the cognitive function of people with epilepsy over time [7], [19], [20], [21], [22]. These discrepancies are likely the result of differing methodologies applied across different research groups.

The exact cause of cognitive decline in people with epilepsy remains unclear and may be reflective of multiple factors, including the underlying etiology of the disease, the neurobiological consequences of seizures, the adverse effects of antiepileptic drug (AED) exposure, and psychosocial dysfunction [1], [2], [3], [4], [5]. Antiepileptic drugs have been considered the principal culprits, with memory, attention, psychomotor speed, and information processing being the cognitive domains most commonly reported to be affected following drug treatment [23]. However, recent work suggesting that some people with epilepsy are cognitively compromised from the time of initial diagnosis would advocate the involvement of more intrinsic biological processes, including epileptogenesis [4], [24]. Genetics may also play a contributory role.

Polymorphic variants in genes encoding neuron-restrictive silencer factor (NRSF) and brain-derived neurotrophic factor (BDNF) are known to be correlated with cognitive ability in the elderly [25], [26], [27], [28]. Neuron-restrictive silencer factor, a transcriptional repressor reported to regulate the expression of more than 2000 genes [29], and its downstream target BDNF, a neuron-specific growth factor involved in neurogenesis, cell survival, and synaptic plasticity [30], [31], [32], [33], have been shown to be differentially regulated in rodent models of epilepsy [34], [35], [36], [37], [38], [39], [40], [41], [42], [43]. The involvement of these two genes in both cognition and epilepsy supports a role for the NRSF–BDNF pathway in epilepsy-associated cognitive dysfunction. Consistent with this pathway being modulated by drug action, several AEDs modify NRSF signaling in neuroblastoma cells [44], [45].

We have undertaken a study of variation in the NRSF and BDNF genes and its association with cognitive function in individuals with newly diagnosed epilepsy and with the change in cognitive function over the first year of treatment following diagnosis. We used a haplotype-tagging approach to assess genetic variation and drew DNA samples and cognitive data from the subgroup analysis of the SANAD trial [3], [4].

## 2 Material and methods

### 2.1 Subjects

A total of 2437 patients were recruited into the SANAD trials (ISRCTN 38354748) [9], [10], of whom 155 were included in the subgroup analysis of cognitive function [3], [4]. Of these, 84 patients had both baseline neuropsychological assessment and a DNA sample, with 70 also having further neuropsychological assessment at approximately 12 months after initial presentation. A comprehensive description of the patient population is provided elsewhere [3], [4], [9], [10]. All subjects were of self-reported Caucasian ancestry and were neurologically normal, MRI negative, and had not previously been treated with any AED. Collection of DNA was approved by the North-West Multicentre Research Ethics Committee in August 2002 (ref: MREC 02/8/45). All patients, or their parents/guardians in the case of minors, provided written informed consent to the use of their DNA and relevant clinical information in this analysis.

### 2.2 Cognitive assessment data

Patients recruited into the SANAD trial were assessed for cognitive function at baseline and during follow-up studies using a neuropsychological test battery designed to assess multiple cognitive domains, including memory, psychomotor speed, information processing, mental flexibility, and mood. The test battery methods are described in detail elsewhere [4]. Only those aspects of the battery that had previously been shown to differ significantly between patients with epilepsy and healthy controls were employed in the genetic association analysis (see Table 1). All 84 subjects contributed to a cross-sectional analysis of genetic influences on baseline cognitive function. The 70 patients who also had a 12-month neuropsychological assessment were additionally included in a longitudinal analysis, investigating the influence of genetic variants on the change in cognitive function from baseline.

### 2.3 Selection of genetic variants

Markers mapping to the NRSF and BDNF genes and their respective flanking sequences (10 kb upstream and downstream) were selected based on implications from the literature and/or maximum genetic coverage through selection of haplotype-tagging SNPs (htSNPs). Haplotype-tagging SNPs were identified using the pairwise-tagging function (r^2^ threshold, 0.8) within Haploview 4.1 software (www.broad.mit.edu/mpg/haploview/) and genotype data corresponding to individuals from the CEPH trios of European descent from HapMap Genome Browser release #28 (August 2010, NCBI build 36, dbSNP b126). Single nucleotide polymorphisms were filtered to include only those with a minor allele frequency (MAF) of greater than 5% within a Caucasian population.

### 2.4 Genotyping

A total of 38 SNPs were selected for genotyping: 14 in NRSF and 24 in BDNF. Multiplex primer assays were designed using Sequenom Assay Design software (https://mysequenom.com/default.aspx). Single nucleotide polymorphisms were divided across two 20-plex assays. Oligonucleotides were purchased from Metabion (Martinsried, Germany). Polymerase chain reaction assays were carried out on a Veriti thermal cycler (Applied Biosciences, Carlsbad, CA, USA) in a 384-well microtiter plate using 20 ng of genomic DNA and with a final reaction volume of 4 μl. As a measure of quality control, six replication samples and six blank controls were used. Genotyping was performed on a MALDI-TOF-based Sequenom iPLEX MassARRAY® platform (Sequenom Inc., San Diego, CA, USA), according to the manufacturer's instructions.

### 2.5 Data analysis

**Fig. 1:**
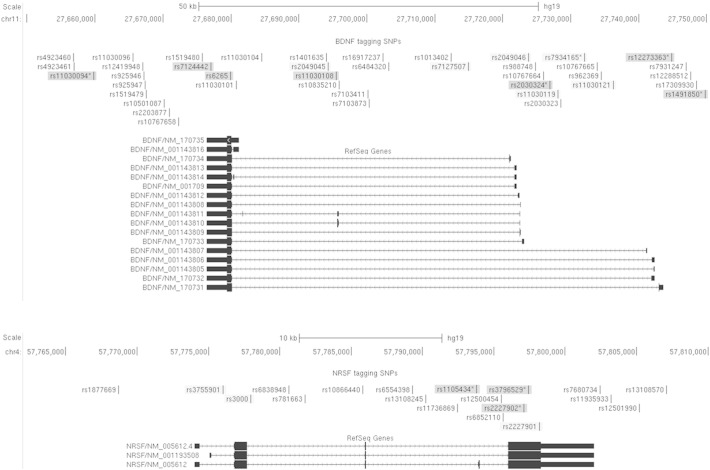
Schematic representation of genotyped haplotype-tagging SNPs (htSNPs) spanning the BDNF (*top*) and NRSF (*bottom*) genes. Highlighted markers represent those selected for genotype analysis; dark gray indicates htSNPs and/or functional SNPs selected for inclusion in the genetic association, and light gray indicates SNPs captured by these selected htSNPs (r^2^ > 0.88) from linkage disequilibrium (LD) analysis of the genotype data. The remaining SNPs represent genetic coverage over the entire locus, including 10 kb flanking sequence, as determined by pairwise-tagging (r^2^ > 0.8, indicating that a pair of SNPs are in strong LD and that one allele at one locus tags another allele at separate locus meaning that only one SNP needs to be genotyped) using HapMap CEU genotype data and Haploview 4.1 software (www.broad.mit.edu/mpg/haploview/). *SNPs shown from genetic analysis to be significantly associated. Image generated using UCSC Genome browser (https://genome.ucsc.edu/).

Descriptive analysis of the patient cohort was carried out using SPSS 22.0 (see Table 2). Cognitive tests used for cross-sectional and longitudinal analyses are listed in Table 1. A total of ten functional and/or nonsynonymous SNPs were selected for the genetic association analysis based on evidence from the literature (references listed in Table 3). These included three SNPs in NRSF (rs1105434, rs2227902, rs3796529) and seven SNPs in BDNF (rs1491850, rs12273363, rs2030324, rs11030108, rs6265, rs7124442, rs11030094). A schematic representation of the genomic coverage of these htSNPs is shown in Fig. 1. To determine associations between cognitive test scores and genotype frequency, regression analysis and expectation–maximization optimization was performed as it accounts for the estimated maximum likelihood of parameters for the longitudinal model [46]. Analysis was based on multilevel mixed-effects linear regression using Restricted Maximum Likelihood (REML) which maximizes the estimated likelihood of variance components affecting the observed measurement (i.e., cognitive test score), invariant to the fixed effects [47]. The REML regression was applied to correct for biases that may have arisen due to selection [48], [49]. To correct for multiple testing, the data were permutated 1000 times. Permutation testing was performed with respect to the number of markers at the gene level. The REML regression and permutation testing were performed using Stata v.9.2. Cognitive data were normally distributed, and age, sex, epilepsy type, number of previous seizures at baseline (continuous variable, cross-sectional analysis), and freedom from seizures since baseline (categorical variable, longitudinal analysis) were accounted for by covarying their effects. All P-values lower than 5% were regarded as significant. Linkage disequilibrium (LD) analysis was performed using the D-prime (D′) statistic which states the normalized covariance for a given pair of markers where a D′ value of 1 represents complete LD. Composite genotype analysis was performed using Golden HelixTree Genetic Analysis software version 5.0 (Golden Helix, Inc., Bozeman, MT, USA).

## 3 Results

### 3.1 Demographic and clinical characteristics

A summary of the study population is provided in Table 2. The mean age of subjects at baseline was 40 years, with a range of 15 to 71 years. There were marginally more females (55%), and the majority of subjects were considered to have focal epilepsy (82%). The mean number of days from baseline assessment to the 12-month follow-up assessment was 388 days, ranging from 350 to 566 days. The number of individuals that were seizure-free for the entire period from baseline until the 12-month assessment was 19.

### 3.2 Association of NRSF and BDNF SNPs with memory related tasks

A total of 36 SNPs were successfully genotyped; these are listed in Table 3. All were in Hardy–Weinberg equilibrium (HWE) and had a MAF > 0.05. Two SNPs (NRSF rs11736869 and BDNF rs11030119) from the original panel were excluded as they had a call rate of less than 95%, the accepted cut-off for genotype-based studies [50]. Two patient samples were excluded from the analysis as they failed quality control checks. Ten SNPs were selected for inclusion in the genetic association analysis on the basis of maximum genetic coverage through the use of htSNPs (Fig. 1) or based on known or proposed functional effects (see *Reference*, Table 3) [25], [26], [27], [51], [52], [53], [54], [55], [56], [57], [58], [59], [60], [61], [62], [63], [64], [65], [66], [67], [68]. These included three SNPs in NRSF (rs1105434, rs2227902, rs3796529) and seven SNPs in BDNF (rs1491850, rs12273363, rs2030324, rs11030108, rs6265, rs7124442, rs11030094).

Regression analysis of cross-sectional cognitive test scores with individual SNP genotypes indicated statistically significant associations for the respective NRSF markers rs1105434 (P = 0.03) and rs2227902 (P = 0.02) with delayed recall, as assessed by the Rey Auditory Verbal Learning Task (AVLT), and serial recall, as assessed by the figure recognition task (Table 4). Three BDNF markers were also associated with the Rey AVLT in the cross-sectional analysis: rs1491850 (P = 0.05, immediate recall), rs11030094 (P = 0.02, delayed recall), and rs2030324 (P = 0.03, immediate recall and P = 0.01, delayed recall) (Table 4). In the longitudinal analysis, a mixed effect REML regression model was used to account for repeated measures and within-subject covariance [47], [48], [49]. After correcting for covariate effects, NRSF rs2227902 was again identified as being significantly (P = 0.01) associated with memory function as was BDNF rs12273363 (P = 0.03) and both in relation to Rey AVLT delayed recall scores. The independent effect of these SNPs in predicting memory function showed only BDNF rs12273363 to be significant (P = 0.04) (Table 5). Psychomotor speed was also found to be significantly affected by genotype (NRSF rs3796529, P = 0.04) in the longitudinal analysis, assessed through visual reaction time (nondominant hand, Table 5). In both the cross-sectional and longitudinal analyses, no significant associations were found between individual SNP genotypes and cognitive test scores measuring information processing (Table 4, Table 5).

### 3.3 Haplotype structure of NRSF and BDNF genes

**Fig. 2:**
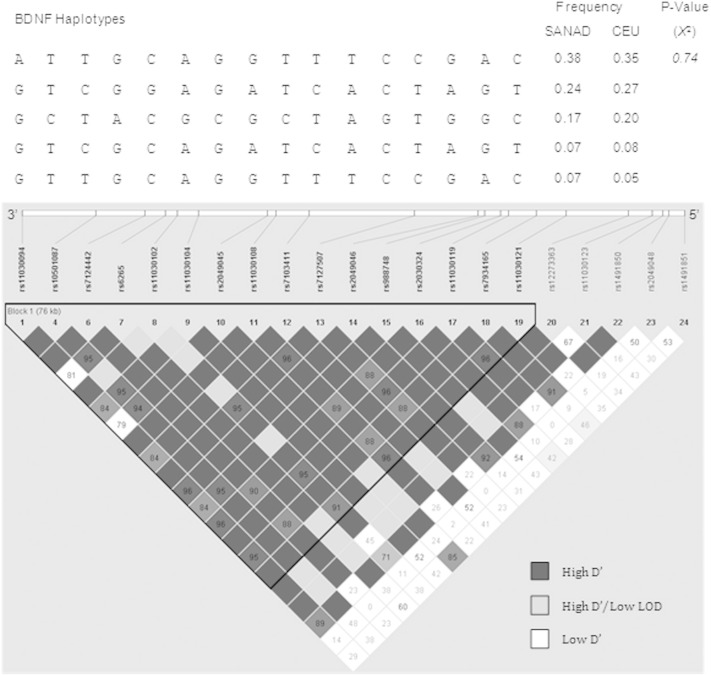
Linkage disequilibrium (LD) and haplotype analysis of BDNF markers in patients with newly diagnosed epilepsy. Haplotype block structure of the BDNF gene indicating strong LD (dark gray squares) based on D′ estimates calculated from 82 individuals with newly diagnosed epilepsy. Haplotype blocks, represented by a black triangular border, were determined using 95% confidence intervals proposed by Gabriel et al. [70] which defined a single block for the BDNF gene. Individual haplotypes making up the BDNF haplotype block are depicted above the LD plot and are compared to haplotype frequencies present in the HapMap CEU cohort. Haplotypes with a minor allele frequency of 0.05 or above were included. Haplotype structure did not significantly differ between the two cohorts (P = 0.74, chi-square test; χ^2^). LOD; log of the likelihood odds ratio, a measure of confidence in the D′ value.

**Fig. 3:**
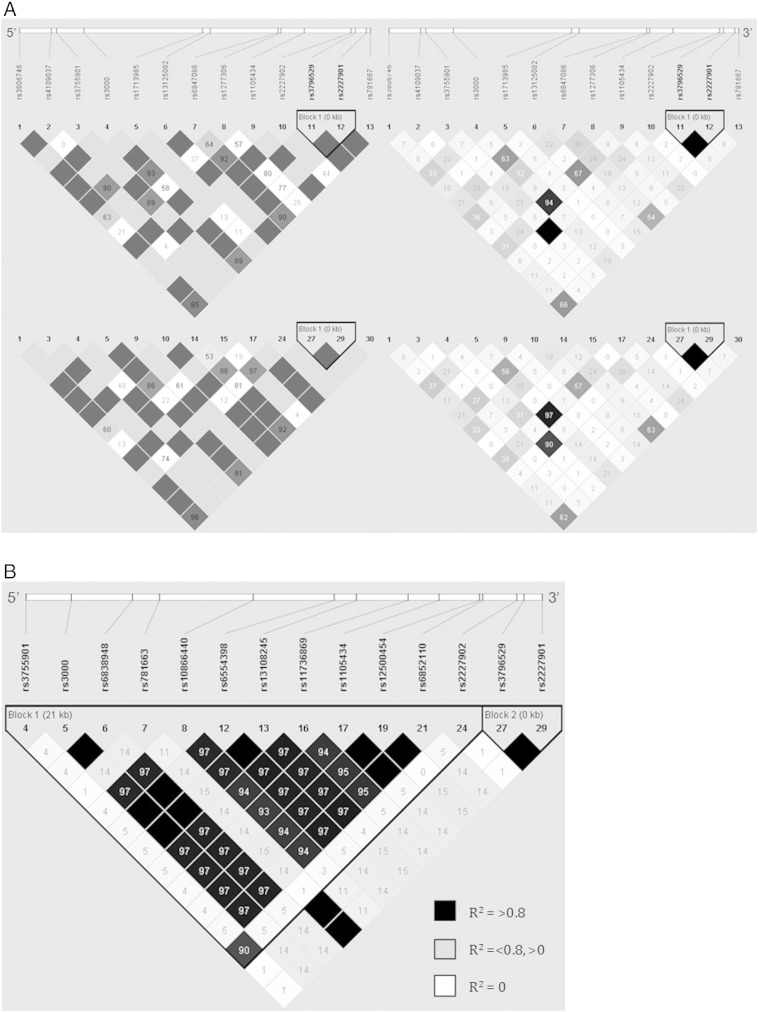
Linkage disequilibrium (LD) and haplotype analysis of NRSF markers in patients with newly diagnosed epilepsy. A, Haplotype block structure of the NRSF gene in the SANAD cohort (top) and the HapMap CEU cohort (bottom) based on D′ (*left*) and r^2^ (*right*) estimates. A similar pattern of LD was observed between the two study cohorts. B, LD analysis in the HapMap CEU cohort using alleles captured through haplotype-tagging indicates two haplotype blocks, represented by black triangular borders, and strong LD over the region. Haplotype blocks were determined using 95% confidence intervals proposed by Gabriel et al. [70].

To ensure that the markers identified from our genetic association were reflective of genetic differences associated with cognitive performance as opposed to differences in ancestry, LD analysis was performed using Lewontin's normalized D′ statistic [69] and compared to LD patterns generated using genotype data from the HapMap CEU cohort as a reference group (Fig. 2, Fig. 3). Haplotype blocks were defined using 95% confidence intervals proposed by Gabriel et al. [70]. Using this method, markers within the BDNF gene were shown to be inherited as a single haplotype block spanning 76 kb, with evidence of recombination within the promoter sequence represented by low D′ values (Fig. 2). Within the NRSF gene, a single haplotype block spanning 774 bp was defined, composed of the rs3796529 and rs2227901 markers (Fig. 3A). Analysis of the genetic coverage over the locus captured by htSNPs selected for inclusion in our genetic association study using genotype data from the HapMap CEU cohort showed an additional haplotype block of strong LD spanning 21 kb (Fig. 3B). Evidence of recombination, depicted by white regions on the LD plot, was observed in the region containing a coding variable number tandem repeat (VNTR) within exon 4 of the NRSF gene that is tagged by the rs2227902 SNP [25] (Fig. 3B), shown from our genetic association to be significantly correlated with memory performance in patients with newly diagnosed epilepsy (Table 4, Table 5).

Pairwise tagging SNP analysis (r^2^ > 0.8) of genotype data revealed that NRSF rs1105434, rs2227902, and rs3796529 were in strong LD with the rs3000 (r^2^ = 0.94), rs3755901 (r^2^ = 1.0), and rs2227901 (r^2^ = 1.0) markers, respectively. For BDNF, the markers rs1491850, rs12273363, rs2030324, rs11030108, rs6265, rs7124442, and rs11030094 represented 13/24 SNPs genotyped for this gene (see Fig. 1 for alleles captured), indicating strong LD across the gene. Linkage disequilibrium patterns across the BDNF and NRSF genes in this modest sample showed no major differences in comparison to LD-plots generated from the HapMap CEU dataset (Fig. 2, Fig. 3A), eliminating the possibility of population stratification in our model.

### 3.4 Association of NRSF–BDNF composite genetic model with Rey AVLT (delayed) scores

**Fig. 4:**
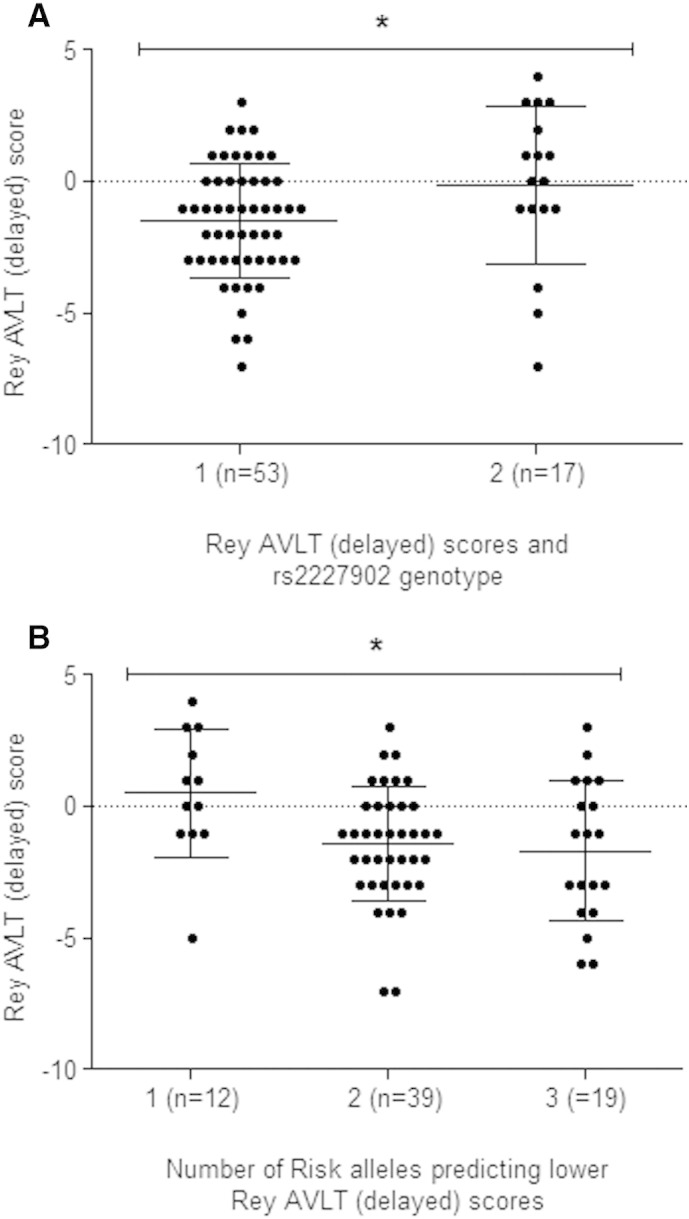
Association of NRSF–BDNF composite-genotype with Rey Auditory Verbal Learning Task (AVLT) delayed recall scores over time. A, Association of NRSF rs2227902 with Rey AVLT delayed recall scores. Group 1 represents individuals homozygous for the wild type risk allele rs2227902 (G); Group 2 represents individuals possessing at least 1 copy of the minor nonrisk allele rs2227902 (T). Horizontal lines represent the mean change with standard deviation from baseline to 12-month reassessment scores. A lower score correlates with a greater reduction in memory performance. A significant decrease in test scores was observed between the two groups (Mann–Whitney test, P = 0.014). B, Risk alleles for NRSF rs2227902 and BDNF rs6265 were grouped and the number of alleles scored as follows: Group 1 represents 0–1 risk alleles, Group 2 represents 2 risk alleles in individuals that were either heterozygous for each SNP or homozygous for rs2227902 (G) and Group 3 represents 3–4 risk alleles. Linear regression analysis showed a significant difference between the groups (P = 0.02).

We have previously shown that the major allele of NRSF rs2227902 (G) is associated with reduced cognitive performance in the elderly [25]. To determine its effect on cognition in adults with newly diagnosed epilepsy, we correlated the number of rs2227902 risk alleles with delayed recall Rey AVLT scores as these were shown to be significantly associated in our longitudinal analysis (P = 0.02, Table 5). Fig. 4A illustrates a similar trend to that observed in the aging population, with individuals homozygous for the wild type (WT) allele deteriorating to a significantly (P = 0.04, unpaired t-test) greater extent than individuals possessing at least one copy of the minor allele in terms of delayed recall in Rey AVLT.

Interaction between the rs2227902 marker and the BDNF rs6265 marker has been described previously [25]. A composite-genotype model was used in order to determine association between the cross-sectional and longitudinal cognitive test scores with the sum of risk alleles for the rs2227902 and rs6265 markers. The WT allele (G), which tags a 5-copy coding VNTR within exon 4 of the NRSF gene, was considered to be the risk allele for rs2227902, whereas the minor allele (Met66-A) was considered the risk allele for rs6265 [25], [27]. Additive-effect linear regression analysis with permutation testing showed that the change in delayed recall in Rey AVLT in the longitudinal analysis was significantly associated with the number of risk alleles (P value = 0.02; Fig. 4B). In our previous report, this interaction was shown to specifically reflect a haplotype containing the rs2227902 (T) marker, which tags a 4-copy variant of the NRSF VNTR, and BDNF rs6265 (Val66-G) [25]. Individuals possessing this haplotype had significantly higher scores of general intelligence than those with one or neither of these variants, or in individuals possessing the NRSF–BDNF ‘risk’ variants. To test the direction of this interaction in terms of risk or nonrisk alleles predicting cognitive performance, linear regression was applied to the different allele groupings as shown in Table 6. Due to the low minor allele frequencies of rs2227902 and rs6265, individuals were grouped by the presence or absence of the minor alleles. Data were controlled for age, sex, and epilepsy type by covarying their effects. Consistent with an additive interaction between the rs2227902 and rs6265 ‘nonrisk’ alleles in determining higher cognitive performance in the elderly, our composite genotype model showed a positive correlation between the presence of the rs2227902 (T)_rs6265 (G) genotype and higher Rey AVLT scores in our epilepsy cohort (Table 6, P = 0.01; beta-coefficient 0.31). No such interaction was observed between the other NRSF–BDNF groupings and cognitive test scores which again supports previous findings [25].

## 4 Discussion

Cognitive dysfunction has been reported in people with newly diagnosed epilepsy. These individuals are naïve to the long-term effects of AED treatment and the cumulative effects of recurrent seizures, suggesting the involvement of other intrinsic and/or environmental factors. In this study, we provide preliminary evidence to suggest that variants within the NRSF and BDNF genes influence cognitive function in adults with newly diagnosed epilepsy at both baseline and over the first year after diagnosis. Genetic effects were specific to memory-related tasks and psychomotor speed (longitudinal analysis). In the cross-sectional analysis, we found significant associations for NRSF rs1105434 and rs2227902 and BDNF rs1491850, rs2030324, and rs11030094, with NRSF rs2227902 and rs3796529 and BDNF rs12273363 implicated in the longitudinal model. These findings are consistent with previous studies showing association between the NRSF and BDNF genes and cognitive function in a healthy aging population and in neurological disorders [58], [71], [72].

All of the SNPs identified in this study have previously been associated with neurological disease or as markers of phenotypic traits associated with CNS dysfunction. For example, the BDNF SNPs rs1491850 and rs11030094, shown in our cross-sectional analysis to be associated with immediate and delayed recall in the Rey AVLT, respectively, have recently been identified as important genetic variants in Alzheimer's disease-related neurodegeneration and cognitive impairments [26], [67]. The BDNF rs1491850 has also been implicated in treatment response phenotypes and remission status in major depressive disorder [54], [55]. We also observed an association between Rey AVLT scores and the BDNF SNP rs2030324 which has previously been associated with cognitive processes in healthy aging [27] and multiple sclerosis [73].

Significant associations with delayed recall performance were also apparent in our longitudinal analysis with respect to NRSF rs2227902 and BDNF rs12273363. This may reflect a distinct regulatory pathway in the modulation of verbal memory in late-onset epilepsy. The location of five of the seven associated SNPs within noncoding regions of the genome is suggestive of a mechanistic role. Several studies have shown enrichment of disease-associated SNPs within tissue-specific enhancers [74], [75], [76], [77], [78]. Polymorphisms within such regulatory elements can alter transcription factor binding motifs and thus the expression of a gene through modulation of signal transduction responses in both a tissue-specific and stimulus-dependent manner. Dysregulation of the BDNF gene is well documented in neurological disorders [56], [79], [80], [81], [82]. Elaborate modulation of BDNF mRNA expression is mediated by nine functional promoters, some of which are influenced by cis-regulatory elements [58], [83]. One such element, BE5.2, which contains the noncoding SNP rs12273363, implicated in memory performance in our longitudinal assessment, has been shown to differentially regulate BDNF promoter 4 activity in a stimulus-inducible, allele-specific, and tissue-dependent manner [58]. This correlates with previous findings of allele-specific differences in pro-BDNF density in postmortem brain tissue in which the minor allele of rs12273363 was associated with reduced hippocampal expression [56]. Association between NRSF rs3796529 and psychomotor speed was also found in our longitudinal study; however, this did not withstand correcting for covariate effects.

The BDNF SNP rs6265 has been extensively studied in the field of cognition, with many publications supporting its role in the regulation of cognitive function. Our study did not find a direct association between rs6265 and cognitive function in patients with epilepsy. However, when we analyzed this SNP in combination with NRSF rs2227902 based on our previous finding of an additive interaction between these two polymorphisms in age-related cognitive function [25], the number of risk alleles was inversely correlated with memory performance (Fig. 4A).

Linear regression analysis of the different groupings of these two SNPs based on the presence or absence of the risk or nonrisk alleles showed that the genetic association was significant in relation to a haplotype containing the nonrisk alleles NRSF rs2227902 (T) and BDNF rs6265 (Val66-G), which correlated with higher Rey AVLT test scores as indicated by the positive beta coefficient value (Table 6). This is consistent with previous findings in the elderly cohort suggesting that this allelic combination may improve cognitive performance or slow the rate of cognitive decay over individuals possessing the proposed risk variants which may predict risk for more rapid cognitive decline over time, as demonstrated in BDNF rs6265 (Met66-A) carriers relative to rs6265 (Val66-G) homozygotes in Alzheimer's disease [84].

Support for the NRSF–BDNF pathway as a potential mechanism in cognitive dysfunction associated with neurological disorders comes from studies on Huntington's disease, where it has been shown that WT but not mutant huntingtin protein regulates BDNF transcription through cytoplasmic sequestering of NRSF [85]. Furthermore, genetic variants of the REST-interacting LIM domain protein (RILP/Prickle-1), an important candidate involved in the nuclear translocation and repressive functioning of NRSF [86], have been associated with autosomal-recessive progressive myoclonus epilepsy–ataxia syndrome, the symptoms of which include seizures and cognitive decline [87]. Epigenetic parameters may also be important in this regulatory network as suggested by interaction of the NRSF-silencing complex with the histone demethylase SMCX, a gene implicated in X-linked mental retardation and epilepsy [88], resulting in chromatin remodeling and downstream regulation of NRSF target genes including BDNF. Other chromatin remodeling proteins associated with this silencing complex have been implicated in memory impairment, including histone deacetylase 2 (HDAC2) [89], [90] and methyl CpG binding protein 2 (MeCP2) which is mutated in Rett syndrome resulting in NRSF/coREST-mediated repression of BDNF expression [91]. Further support comes from evidence that glycolytic inhibitor 2-deoxy-D-glucose modulation of the NRSF–CBP (C-terminal binding protein) complex enhances the repressive chromatin environment surrounding the BDNF gene, consequently blocking epileptogenesis [37], [40]. In addition, investigations into functional abnormalities observed in patients with Korsakoff's syndrome, a neurological disorder caused by thiamine deficiency, found a strong correlation between reduced glycolysis and delayed memory performance [92]. Both NRSF and BDNF have been linked to impairments of neurogenesis in this disorder [93]. Collectively these studies support a role for dysregulation of the NRSF–BDNF pathway in cognitive decline associated with neurological disease.

## 5 Conclusion

Our data support a trend towards association of polymorphic variants within the NRSF and BDNF genes and memory-related tasks in patients with a new diagnosis of epilepsy. These associations reached statistical significance in both the cross-sectional and longitudinal assessment suggesting the influence of genetic background on the susceptibility to memory decline in adults with new onset epilepsy. Our findings are consistent with previous literature in the field but should be considered with caution, not least because of the small sample size. In addition, we were unable to account for potentially confounding variables including the influence of AED exposure or possible practice effects associated with repeat application of cognitive tests. Although they require replication in a larger study sample, these observations lend weight to the known involvement of NRSF–BDNF markers in the modulation of cognitive performance in healthy aging and also in neurological and psychiatric disorders.

## Role of funder

This study was supported by the UK Biotechnology and Biological Sciences Research Council (BBSRC) in the form of studentship funding to AW (ref. BB/F016905/1). The funder had no involvement in study design, collection, analysis or interpretation of data, writing the article, or in the decision to submit the article for publication.

## Figures and Tables

**Table 1:** Selected cognitive tests employed in this analysis.

Analysis	Domain	Test	Measured variable
Cross-sectional	Memory	Figure recognition (serial task)	Number of figures correctly identified in the serial task
Rey Auditory Verbal Learning Task, AVLT (immediate and delayed)	Sum of words recalled over the 5 trials and the number of words recalled following a 30-minute delay
Story recall (immediate)	Number of story units recalled immediately and following a 10-minute delay
Psychomotor speed	Finger tapping (dominant hand)	Average number of taps for the dominant hand across five trials
Adult Memory and Information Processing (average speed)	Average number of digits crossed out over two trials
Longitudinal	Memory	Rey Auditory Verbal Learning Task, AVLT (immediate and delayed)	Sum of words recalled over the 5 trials and the number of words recalled following a 30-minute delay
Psychomotor speed	Visual reaction time, VRT (nondominant hand)	Average reaction time (min/s) for the dominant and nondominant hand
Information processing	Computerized Visual Search Task, CVST	Average speed of response (seconds)

Cognitive tests selected based on aspects of the battery previously shown to significantly differ between patients with epilepsy and healthy controls [4].

**Table 2:** Demographic and clinical profile of the study cohort at baseline and 12-month assessment.

Variable		Baseline (n = 82)	12 months (n = 70)
Sex	Males (n)	37	31
Females (n)	45	39
Age	Mean [range]	40 [15–71]	42 [16–70]
Epilepsy type	Generalized (n)	15	13
Focal (n)	67	57
No. of previous seizures at baseline	Mean [range]	112 [2–3300]	–
Remission status at follow-up	Seizure-free (n)	–	19

**Table 3:** Minor allele frequencies and Hardy–Weinberg equilibrium of NRSF and BDNF SNPs.

Gene	Marker	Chromosomal position	Base pair change (major>minor allele)	Genotype distribution	HWE P-value	MAF	Reference
NRSF	rs3806746	57773330	A>G	25/40/6	0.07	0.37	
rs4109037	57775609	A>T	65/17/0	0.30	0.10	
rs3755901	57775996	A>T	64/17/1	0.91	0.12	
rs3000	57777945	C>T	27/36/9	0.57	0.38	
rs1713985	57786450	A>C	68/14/0	0.40	0.09	
rs13125082	57787000	T>G	38/27/6	0.70	0.28	
rs6847086	57791864	G>A	25/36/9	0.48	0.39	
rs1277306	57792078	T>C	35/40/7	0.34	0.33	
**rs1105434**	57793751	G>A	29/33/9	0.93	0.36	[51]
**rs2227902**	57797100	G>T	57/15/0	0.32	0.10	[25]
**rs3796529**	57797414	G>A	49/26/2	0.50	0.20	[25], [52]
rs2227901	57798189	G>A	54/26/2	0.58	0.18	
rs781667	57798469	T>C	40/38/4	0.18	0.28	
BDNF	rs1491851	27752763	C>T	33/27/12	0.13	0.35	
rs2049048	27750586	C>T	47/18/4	0.22	0.19	
**rs1491850**	27749725	T>C	24/44/14	0.42	0.44	[26], [53], [54], [55]
rs11030123	27748285	G>A	65/14/2	0.24	0.11	
**rs12273363**	27744859	T>C	45/27/0	0.05	0.19	[53], [55], [56], [57], [58]
rs11030121	27736207	C>T	34/28/9	0.40	0.32	
rs7934165	27731983	A>G	16/35/14	0.53	0.49	
**rs2030324**	27726915	T>C	25/37/16	0.73	0.44	[27], [57], [73]
rs988748	27724745	C>G	50/28/3	0.70	0.21	
rs2049046	27723775	A>T	28/35/18	0.27	0.44	
rs7127507	27714884	T>C	34/27/11	0.16	0.34	
rs7103411	27700125	T>C	43/27/2	0.35	0.22	
**rs11030108**	27695464	G>A	38/31/11	0.26	0.33	[26]
rs2049045	27694241	G>C	55/25/2	0.67	0.18	
rs11030104	27684517	A>G	51/28/3	0.72	0.21	
rs11030102	27681596	C>G	43/34/3	0.23	0.25	
**rs6265**	27679916	G>A	53/27/2	0.50	0.19	[25], [27], [59], [60], [61], [62], [63], [64], [65], [66]
**rs7124442**	27677041	T>C	38/31/13	0.13	0.35	[59]
rs4923463	27672500	A>G	52/27/3	0.83	0.20	
rs10501087	27670108	T>C	44/26/2	0.42	0.21	
rs7927728	27667472	G>A	60/8/1	0.25	0.07	
rs11602246	27660926	C>G	63/9/0	0.57	0.06	
**rs11030094**	27659775	G>A	28/33/11	0.80	0.38	[26], [67]

Markers in bold font represent the 10 SNPs selected for further analysis. Genotype distribution represents AA/Aa/aa, where ‘A’ is the wild type allele and ‘a’ the variant allele. Abbreviations: HWE, Hardy–Weinberg equilibrium; htSNPs, haplotype-tagging single nucleotide polymorphisms; MAF, minor allele frequency.

**Table 4:** Genetic association analysis of cross-sectional cognitive data using a regression model adjusted for age, sex, epilepsy type, and number of previous seizures at baseline.

Gene	SNP	Cognitive test	β	Adjusted P-value^a^	95% CI	
Lower	Upper
NRSF	rs1105434	Finger tapping (dominant hand)	− 0.98	0.61	− 4.53	2.57
Story recall (immediate)	0.64	0.22	− 0.43	1.71
Figure recognition (serial)	1.31	0.07	− 0.22	2.83
Rey AVLT (immediate)	1.78	0.23	− 1.27	4.82
Rey AVLT (delayed)	1.00	**0.03**⁎	0.04	2.00
AMIPB average speed	− 3.57	0.06	− 7.37	0.24
rs2227902	Finger tapping (dominant hand)	− 1.78	0.52	− 7.63	4.06
Story recall (immediate)	0.26	0.74	− 1.48	2.00
Figure recognition (serial)	− 2.63	**0.02**⁎	− 5.06	− 0.19
Rey AVLT (immediate)	1.52	0.55	− 3.42	6.46
Rey AVLT (delayed)	0.20	0.83	− 1.80	1.41
AMIPB average speed	− 0.93	0.76	− 7.16	5.31
rs3796529	Finger tapping (dominant hand)	1.19	0.58	− 2.90	5.28
Story recall (immediate)	− 0.50	0.42	− 1.69	0.69
Figure recognition (serial)	0.85	0.36	− 0.93	2.62
Rey AVLT (immediate)	− 1.20	0.49	− 4.73	2.34
Rey AVLT (delayed)	− 0.48	0.40	− 1.63	0.68
AMIPB average speed	0.73	0.78	− 3.86	5.32
BDNF	rs1491850	Finger tapping (dominant hand)	− 1.76	0.28	− 5.06	1.53
Story recall (immediate)	0.44	0.37	− 0.51	1.38
Figure recognition (serial)	0.71	0.28	− 0.66	2.08
Rey AVLT (immediate)	2.81	**0.05**⁎	0.11	5.51
Rey AVLT (delayed)	0.56	0.20	− 0.32	1.44
AMIPB average speed	− 2.78	0.12	− 6.30	0.73
rs12273363	Finger tapping (dominant hand)	− 3.96	0.12	− 8.69	− 0.76
Story recall (immediate)	0.82	0.25	− 0.62	2.25
Figure recognition (serial)	0.21	0.84	− 1.88	2.30
Rey AVLT (immediate)	2.62	0.20	− 1.46	6.71
Rey AVLT (delayed)	0.55	0.42	− 0.78	1.88
AMIPB average speed	− 1.32	0.60	− 6.53	3.90
rs2030324	Finger tapping (dominant hand)	0.33	0.85	− 2.87	3.53
Story recall (immediate)	− 0.50	0.29	− 1.42	0.43
Figure recognition (serial)	− 1.08	0.09	− 2.35	0.19
Rey AVLT (immediate)	− 2.78	**0.03**⁎	− 5.43	− 0.13
Rey AVLT (delayed)	− 1.19	**0.01**⁎	− 2.01	− 0.36
AMIPB average speed	0.96	0.60	− 2.54	4.46
rs11030108	Finger tapping (dominant hand)	− 1.49	0.34	− 4.68	1.70
Story recall (immediate)	0.59	0.19	− 0.30	1.47
Figure recognition (serial)	0.61	0.34	− 0.69	1.90
Rey AVLT (immediate)	2.57	0.06	− 0.02	5.17
Rey AVLT (delayed)	0.74	0.09	− 0.10	1.57
AMIPB average speed	1.10	0.53	− 2.26	4.47
rs6265	Finger tapping (dominant hand)	0.91	0.67	− 3.19	5.00
Story recall (immediate)	− 0.31	0.61	− 1.49	0.88
Figure recognition (serial)	0.32	0.71	− 1.37	2.01
Rey AVLT (immediate)	0.89	0.60	− 2.56	4.35
Rey AVLT (delayed)	0.33	0.58	− 0.78	1.43
AMIPB average speed	− 1.14	0.07	− 8.48	0.20
rs7124442	Finger tapping (dominant hand)	− 1.62	0.29	− 4.65	1.40
Story recall (immediate)	0.44	0.33	− 0.43	1.30
Figure recognition (serial)	0.45	0.48	− 0.80	1.69
Rey AVLT (immediate)	2.34	0.06	− 0.13	4.81
Rey AVLT (delayed)	0.69	0.08	− 0.11	1.48
AMIPB average speed	1.02	0.53	− 2.19	4.23
rs11030094	Finger tapping (dominant hand)	2.57	0.16	− 0.87	6.00
Story recall (immediate)	− 0.36	0.48	− 1.42	0.69
Figure recognition (serial)	− 1.30	0.07	− 2.79	0.20
Rey AVLT (immediate)	− 2.79	0.05	− 5.73	0.15
Rey AVLT (delayed)	− 1.01	**0.02**⁎	− 2.02	− 0.13
AMIPB average speed	2.62	0.15	− 1.21	6.47

Negative β values indicate lower test scores for each copy of the minor allele. Abbreviations: AMIPB, Adult Memory and Information Processing Battery; AVLT, Auditory Verbal Learning Task; β, beta coefficient; CI, confidence interval.

a Permutation testing for the number of markers at the gene level.

⁎ P ≤ 0.05.

**Table 5:** Genetic association analysis of longitudinal cognitive data using a mixed-effect REML regression model adjusted for age, sex, epilepsy type, and remission status at 12-month follow-up (seizure-free or not).

Gene	SNP	Cognitive test	β	P-value	Adjusted P-value^a^	95% CI	
Lower	Upper
NRSF	rs1105434	VRT (nondominant hand)^b^	0.06	0.64	0.70	− 0.21	0.34
CVST^b^	− 0.01	0.95	0.85	− 0.36	0.34
Rey AVLT (immediate)	0.52	0.91	0.90	− 8.20	9.25
Rey AVLT (delayed)	0.12	0.94	0.74	− 2.74	2.98
rs2227902	VRT (nondominant hand)^b^	− 0.07	0.68	0.96	− 0.43	0.28
CVST^b^	0.04	0.84	0.89	− 0.42	0.52
Rey AVLT (immediate)	− 6.68	0.23	0.08	− 17.50	4.14
Rey AVLT (delayed)	3.53	0.08	**0.02**⁎	− 7.49	0.42
rs3796529	VRT (nondominant hand)^b^	0.36	**0.04**⁎	0.08	0.02	0.71
CVST^b^	− 0.25	0.29	0.29	− 0.71	0.21
Rey AVLT (immediate)	2.23	0.68	0.38	− 8.30	12.77
Rey AVLT (delayed)	− 1.29	0.53	0.94	− 5.28	2.69
BDNF	rs1491850	VRT (nondominant hand)^b^	0.15	0.26	0.43	− 0.11	0.40
CVST^b^	0.17	0.27	0.73	− 0.14	0.48
Rey AVLT (immediate)	− 2.33	0.58	0.43	− 10.52	5.86
Rey AVLT (delayed)	− 1.81	0.19	0.08	− 4.55	0.92
rs12273363	VRT (nondominant hand)^b^	0.09	0.59	0.69	− 0.23	0.41
CVST^b^	0.25	0.24	0.45	− 0.16	0.65
Rey AVLT (immediate)	− 8.15	0.12	0.07	− 18.28	1.97
Rey AVLT (delayed)	− 3.99	**0.04**⁎	**0.01**⁎	− 7.74	− 0.25
rs2030324	VRT (nondominant hand)^b^	0.08	0.52	0.31	− 0.17	0.34
CVST^b^	− 0.13	0.39	0.36	− 0.45	0.18
Rey AVLT (immediate)	− 3.80	0.32	0.43	− 11.35	3.75
Rey AVLT (delayed)	− 1.10	0.43	0.84	− 3.80	1.60
rs11030108	VRT (nondominant hand)^b^	0.05	0.44	0.88	− 0.18	0.28
CVST^b^	− 0.00	0.98	0.90	− 0.30	0.29
Rey AVLT (immediate)	− 0.73	0.83	0.51	− 7.35	5.89
Rey AVLT (delayed)	− 0.58	0.66	0.33	− 3.12	1.97
rs6265	VRT (nondominant hand)^b^	0.06	0.69	0.97	− 0.23	0.35
CVST^b^	− 0.02	0.93	0.53	− 0.39	0.36
Rey AVLT (immediate)	1.03	0.84	0.64	− 8.77	10.82
Rey AVLT (delayed)	− 0.46	0.81	0.86	− 4.08	3.16
rs7124442	VRT (nondominant hand)^b^	0.08	0.48	0.61	− 0.14	0.30
CVST^b^	− 0.02	0.92	0.88	− 0.29	0.26
Rey AVLT (immediate)	− 1.39	0.71	0.46	− 8.62	5.84
Rey AVLT (delayed)	− 0.37	0.78	0.48	− 3.01	2.26
rs11030094	VRT (nondominant hand)^b^	0.21	0.13	0.05	− 0.06	0.48
CVST^b^	− 0.09	0.61	0.69	− 0.45	0.27
Rey AVLT (immediate)	− 6.44	0.18	0.19	− 15.76	2.87
Rey AVLT (delayed)	− 1.86	0.24	0.47	− 4.95	1.22

Negative β values indicate lower test scores for each copy of the minor allele. AVLT, Auditory Verbal Learning Task; β, beta coefficient; CI, confidence interval; CVST, Computerized Visual Search Task; REML, Restricted Maximum Likelihood; VRT, visual reaction time.

a Corrected for significant covariate effects (age).

b Analysis undertaken on log-transformed data.

⁎ P < 0.05.

**Table 6:** Association of NRSF–BDNF composite-genotype with Rey Auditory Verbal Learning Task (AVLT) delayed recall scores over time.

NRSF–BDNF haplotype	N	Frequency	β	P-value^a^
rs2227902 (G)_rs6265 (G)	35	50.0	− 0.12	0.31
rs2227902 (G)_rs6265 (A)	18	25.7	− 0.13	0.30
rs2227902 (T)_rs6265 (G)	12	17.1	0.31	**0.01**⁎
rs2227902 (T)_rs6265 (A)	5	7.1	− 0.01	0.97

Major allele of NRSF, rs2227902 (G), and minor allele of BDNF, rs6265 (A), were considered risk alleles. Negative β scores indicate that the presence of risk alleles (or absence of nonrisk alleles) correlates with lower test scores. Positive β scores indicate that the presence of nonrisk alleles (or absence of risk alleles) correlates with higher test scores. Abbreviations: AVLT, Auditory Verbal Learning Task; β, beta coefficient.

a Linear regression model for association between the NRSF marker rs2227902 and the BDNF marker rs6265 with Rey AVLT delayed recall scores over time.

⁎ P < 0.05.

## References

[1] Motamedi G., Meador K. (2003). Epilepsy and cognition. Epilepsy Behav.

[2] Tellez-Zenteno J.F., Patten S.B., Jette N., Williams J., Wiebe S. (2007). Psychiatric comorbidity in epilepsy: a population-based analysis. Epilepsia.

[3] Taylor J., Baker G.A. (2010). Newly diagnosed epilepsy: cognitive outcome at 5 years. Epilepsy Behav.

[4] Taylor J., Kolamunnage-Dona R., Marson A.G., Smith P.E., Aldenkamp A.P., Baker G.A. (2010). Patients with epilepsy: cognitively compromised before the start of antiepileptic drug treatment?. Epilepsia.

[5] Berg A.T. (2011). Epilepsy, cognition, and behavior: the clinical picture. Epilepsia.

[6] Baker G.A., Taylor J., Aldenkamp A.P. (2011). Newly diagnosed epilepsy: cognitive outcome after 12 months. Epilepsia.

[7] Aikia M., Kalviainen R., Riekkinen P. (1999). Five-year follow-up of cognitive performance of adult patients with well-controlled partial epilepsy. Epilepsia.

[8] Aikia M., Salmenpera T., Partanen K., Kalviainen R. (2001). Verbal memory in newly diagnosed patients and patients with chronic left temporal lobe epilepsy. Epilepsy Behav.

[9] Marson A.G., Al-Kharusi A.M., Alwaidh M., Appleton R., Baker G.A., Chadwick D.W. (2007). The SANAD study of effectiveness of carbamazepine, gabapentin, lamotrigine, oxcarbazepine, or topiramate for treatment of partial epilepsy: an unblinded randomised controlled trial. Lancet.

[10] Marson A.G., Al-Kharusi A.M., Alwaidh M., Appleton R., Baker G.A., Chadwick D.W. (2007). The SANAD study of effectiveness of valproate, lamotrigine, or topiramate for generalised and unclassifiable epilepsy: an unblinded randomised controlled trial. Lancet.

[11] Meador K.J. (2002). Cognitive outcomes and predictive factors in epilepsy. Neurology.

[12] Jokeit H., Ebner A. (1999). Long term effects of refractory temporal lobe epilepsy on cognitive abilities: a cross sectional study. J Neurol Neurosurg Psychiatry.

[13] Kent G.P., Schefft B.K., Howe S.R., Szaflarski J.P., Yeh H.S., Privitera M.D. (2006). The effects of duration of intractable epilepsy on memory function. Epilepsy Behav.

[14] Helmstaedter C., Kurthen M., Lux S., Johanson K., Quiske A., Schramm J. (2000). Temporal lobe epilepsy: longitudinal clinical, neuropsychological and psychosocial follow-up of surgically and conservatively managed patients. Nervenarzt.

[15] Helmstaedter C., Kurthen M., Lux S., Reuber M., Elger C.E. (2003). Chronic epilepsy and cognition: a longitudinal study in temporal lobe epilepsy. Ann Neurol.

[16] Andersson-Roswall L., Engman E., Samuelsson H., Sjoberg-Larsson C., Malmgren K. (2004). Verbal memory decline and adverse effects on cognition in adult patients with pharmacoresistant partial epilepsy: a longitudinal controlled study of 36 patients. Epilepsy Behav.

[17] Thompson P.J., Duncan J.S. (2005). Cognitive decline in severe intractable epilepsy. Epilepsia.

[18] Hermann B.P., Seidenberg M., Dow C., Jones J., Rutecki P., Bhattacharya A. (2006). Cognitive prognosis in chronic temporal lobe epilepsy. Ann Neurol.

[19] Piazzini A., Turner K., Chifari R., Morabito A., Canger R., Canevini M.P. (2006). Attention and psychomotor speed decline in patients with temporal lobe epilepsy: a longitudinal study. Epilepsy Res.

[20] Holmes M.D., Dodrill C.B., Wilkus R.J., Ojemann L.M., Ojemann G.A. (1998). Is partial epilepsy progressive? Ten-year follow-up of EEG and neuropsychological changes in adults with partial seizures. Epilepsia.

[21] Helmstaedter C., Elger C.E. (2009). Chronic temporal lobe epilepsy: a neurodevelopmental or progressively dementing disease?. Brain.

[22] Dodrill C.B., Wilensky A.J. (1992). Neuropsychological abilities before and after 5 years of stable antiepileptic drug therapy. Epilepsia.

[23] Park S.P., Kwon S.H. (2008). Cognitive effects of antiepileptic drugs. J Clin Neurol.

[24] Hermann B., Jones J., Sheth R., Dow C., Koehn M., Seidenberg M. (2006). Children with new-onset epilepsy: neuropsychological status and brain structure. Brain.

[25] Miyajima F., Quinn J.P., Horan M., Pickles A., Ollier W.E., Pendleton N. (2008). Additive effect of BDNF and REST polymorphisms is associated with improved general cognitive ability. Genes Brain Behav.

[26] Honea R.A., Cruchaga C., Perea R.D., Saykin A.J., Burns J.M., Weinberger D.R. (2013). Characterizing the role of brain derived neurotrophic factor genetic variation in Alzheimer's disease neurodegeneration. PLoS One.

[27] Miyajima F., Ollier W., Mayes A., Jackson A., Thacker N., Rabbitt P. (2008). Brain-derived neurotrophic factor polymorphism Val66Met influences cognitive abilities in the elderly. Genes Brain Behav.

[28] Voineskos A.N., Lerch J.P., Felsky D., Shaikh S., Rajji T.K., Miranda D. (2011). The brain-derived neurotrophic factor Val66Met polymorphism and prediction of neural risk for Alzheimer disease. Arch Gen Psychiatry.

[29] Bruce A.W., Krejci A., Ooi L., Deuchars J., Wood I.C., Dolezal V. (2006). The transcriptional repressor REST is a critical regulator of the neurosecretory phenotype. J Neurochem.

[30] Huang E.J., Reichardt L.F. (2001). Neurotrophins: roles in neuronal development and function. Annu Rev Neurosci.

[31] Scharfman H., Goodman J., Macleod A., Phani S., Antonelli C., Croll S. (2005). Increased neurogenesis and the ectopic granule cells after intrahippocampal BDNF infusion in adult rats. Exp Neurol.

[32] Pencea V., Bingaman K.D., Wiegand S.J., Luskin M.B. (2001). Infusion of brain-derived neurotrophic factor into the lateral ventricle of the adult rat leads to new neurons in the parenchyma of the striatum, septum, thalamus, and hypothalamus. J Neurosci.

[33] McAllister A.K., Katz L.C., Lo D.C. (1999). Neurotrophins and synaptic plasticity. Annu Rev Neurosci.

[34] Palm K., Belluardo N., Metsis M., Timmusk T. (1998). Neuronal expression of zinc finger transcription factor REST/NRSF/XBR gene. J Neurosci.

[35] Calderone A., Jover T., Noh K.M., Tanaka H., Yokota H., Lin Y. (2003). Ischemic insults derepress the gene silencer REST in neurons destined to die. J Neurosci.

[36] Spencer E.M., Chandler K.E., Haddley K., Howard M.R., Hughes D., Belyaev N.D. (2006). Regulation and role of REST and REST4 variants in modulation of gene expression in in vivo and in vitro in epilepsy models. Neurobiol Dis.

[37] Hu X.L., Cheng X., Cai L., Tan G.H., Xu L., Feng X.Y. (2011). Conditional deletion of NRSF in forebrain neurons accelerates epileptogenesis in the kindling model. Cereb Cortex.

[38] Quinn J.P., Bubb V.J., Marshall-Jones Z.V., Coulson J.M. (2002). Neuron restrictive silencer factor as a modulator of neuropeptide gene expression. Regul Pept.

[39] Roopra A., Huang Y., Dingledine R. (2001). Neurological disease: listening to gene silencers. Mol Interv.

[40] Garriga-Canut M., Schoenike B., Qazi R., Bergendahl K., Daley T.J., Pfender R.M. (2006). 2-Deoxy-D-glucose reduces epilepsy progression by NRSF–CtBP-dependent metabolic regulation of chromatin structure. Nat Neurosci.

[41] Liu M., Sheng Z., Cai L., Zhao K., Tian Y., Fei J. (2012). Neuronal conditional knockout of NRSF decreases vulnerability to seizures induced by pentylenetetrazol in mice. Acta Biochim Biophys Sin (Shanghai).

[42] Ballarín M., Ernfors P., Lindefors N., Persson H. (1991). Hippocampal damage and kainic acid injection induce a rapid increase in mRNA for BDNF and NGF in the rat brain. Exp Neurol.

[43] Nibuya M., Morinobu S., Duman R.S. (1995). Regulation of BDNF and trkB mRNA in rat brain by chronic electroconvulsive seizure and antidepressant drug treatments. J Neurosci.

[44] Gillies S.G., Haddley K., Vasiliou S.A., Jacobson G.M., von Mentzer B., Bubb V.J. (2011). Distinct gene expression profiles directed by the isoforms of the transcription factor neuron-restrictive silencer factor in human SK-N-AS neuroblastoma cells. J Mol Neurosci.

[45] Gillies S., Haddley K., Vasiliou S., Bubb V.J., Quinn J.P. (2009). The human neurokinin B gene, TAC3, and its promoter are regulated by Neuron Restrictive Silencing Factor (NRSF) transcription factor family. Neuropeptides.

[46] Dempster A.P., Laird N.M., Rubin D.B. (1977). Maximum likelihood from incomplete data via the EM algorithm. J R Stat Soc.

[47] Patterson H.D., Thompson R. (1971). Recovery of inter-block information when block sizes are unequal. Biometrika.

[48] Henderson C.R. (1986). Recent developments in variance and covariance estimation. J Anim Sci.

[49] Searle S.R. (1989). Variance components — some history and a summary account of estimation methods. J Anim Breed Genet.

[50] Edenberg H.J., Liu Y. (2009). Laboratory methods for high-throughput genotyping. Cold Spring Harb Protoc.

[51] Kahrizi K., Najmabadi H., Kariminejad R., Jamali P., Malekpour M., Garshasbi M. (2009). An autosomal recessive syndrome of severe mental retardation, cataract, coloboma and kyphosis maps to the pericentromeric region of chromosome 4. Eur J Hum Genet.

[52] Tsolaki M. (2014). Clinical workout for the early detection of cognitive decline and dementia. Eur J Clin Nutr.

[53] Nishimura K., Nakamura K., Anitha A., Yamada K., Tsujii M., Iwayama Y. (2007). Genetic analyses of the brain-derived neurotrophic factor (BDNF) gene in autism. Biochem Biophys Res Commun.

[54] Kocabas N.A., Antonijevic I., Faghel C., Forray C., Kasper S., Lecrubier Y. (2011). Brain-derived neurotrophic factor gene polymorphisms: influence on treatment response phenotypes of major depressive disorder. Int Clin Psychopharmacol.

[55] Gratacos M., Soria V., Urretavizcaya M., Gonzalez J.R., Crespo J.M., Bayes M. (2008). A brain-derived neurotrophic factor (BDNF) haplotype is associated with antidepressant treatment outcome in mood disorders. Pharmacogenomics J.

[56] Dunham J.S., Deakin J.F., Miyajima F., Payton A., Toro C.T. (2009). Expression of hippocampal brain-derived neurotrophic factor and its receptors in Stanley consortium brains. J Psychiatr Res.

[57] Liu L., Foroud T., Xuei X., Berrettini W., Byerley W., Coryell W. (2008). Evidence of association between brain-derived neurotrophic factor gene and bipolar disorder. Psychiatr Genet.

[58] Hing B., Davidson S., Lear M., Breen G., Quinn J., McGuffin P. (2012). A polymorphism associated with depressive disorders differentially regulates brain derived neurotrophic factor promoter IV activity. Biol Psychiatry.

[59] Mercader J.M., Ribases M., Gratacos M., Gonzalez J.R., Bayes M., de Cid R. (2007). Altered brain-derived neurotrophic factor blood levels and gene variability are associated with anorexia and bulimia. Genes Brain Behav.

[60] Hall D., Dhilla A., Charalambous A., Gogos J.A., Karayiorgou M. (2003). Sequence variants of the brain-derived neurotrophic factor (BDNF) gene are strongly associated with obsessive–compulsive disorder. Am J Hum Genet.

[61] Lang U.E., Hellweg R., Kalus P., Bajbouj M., Lenzen K.P., Sander T. (2005). Association of a functional BDNF polymorphism and anxiety-related personality traits. Psychopharmacology (Berl).

[62] Jiang X., Xu K., Hoberman J., Tian F., Marko A.J., Waheed J.F. (2005). BDNF variation and mood disorders: a novel functional promoter polymorphism and Val66Met are associated with anxiety but have opposing effects. Neuropsychopharmacology.

[63] Egan M.F., Kojima M., Callicott J.H., Goldberg T.E., Kolachana B.S., Bertolino A. (2003). The BDNF val66met polymorphism affects activity-dependent secretion of BDNF and human memory and hippocampal function. Cell.

[64] Hariri A.R., Goldberg T.E., Mattay V.S., Kolachana B.S., Callicott J.H., Egan M.F. (2003). Brain-derived neurotrophic factor val66met polymorphism affects human memory-related hippocampal activity and predicts memory performance. J Neurosci.

[65] Lin Y., Cheng S., Xie Z., Zhang D. (2014). Association of rs6265 and rs2030324 polymorphisms in brain-derived neurotrophic factor gene with Alzheimer's disease: a meta-analysis. PLoS One.

[66] Cheah S.Y., Lawford B.R., Young R.M., Connor J.P., Phillip Morris C., Voisey J. (2014). BDNF SNPs are implicated in comorbid alcohol dependence in schizophrenia but not in alcohol-dependent patients without schizophrenia. Alcohol Alcohol.

[67] Laumet G., Chouraki V., Grenier-Boley B., Legry V., Heath S., Zelenika D. (2010). Systematic analysis of candidate genes for Alzheimer's disease in a French, genome-wide association study. J Alzheimers Dis.

[68] Zai C.C., Manchia M., De Luca V., Tiwari A.K., Squassina A., Zai G.C. (2010). Association study of BDNF and DRD3 genes in schizophrenia diagnosis using matched case–control and family based study designs. Prog Neuropsychopharmacol Biol Psychiatry.

[69] Lewontin R.C. (1964). The interaction of selection and linkage. I. General considerations; heterotic models. Genetics.

[70] Gabriel S.B., Schaffner S.F., Nguyen H., Moore J.M., Roy J., Blumenstiel B. (2002). The structure of haplotype blocks in the human genome. Science.

[71] Lu T., Aron L., Zullo J., Pan Y., Kim H., Chen Y. (2014). REST and stress resistance in ageing and Alzheimer's disease. Nature.

[72] Laske C., Stellos K., Hoffmann N., Stransky E., Straten G., Eschweiler G.W. (2011). Higher BDNF serum levels predict slower cognitive decline in Alzheimer's disease patients. Int J Neuropsychopharmacol.

[73] Weinstock-Guttman B., Benedict R.H., Tamano-Blanco M., Ramasamy D.P., Stosic M., Polito J. (2011). The rs2030324 SNP of brain-derived neurotrophic factor (BDNF) is associated with visual cognitive processing in multiple sclerosis. Pathophysiology.

[74] Bhandare R., Schug J., Le Lay J., Fox A., Smirnova O., Liu C. (2010). Genome-wide analysis of histone modifications in human pancreatic islets. Genome Res.

[75] Ward L.D., Kellis M. (2012). HaploReg: a resource for exploring chromatin states, conservation, and regulatory motif alterations within sets of genetically linked variants. Nucleic Acids Res.

[76] Ernst J., Kheradpour P., Mikkelsen T.S., Shoresh N., Ward L.D., Epstein C.B. (2011). Mapping and analysis of chromatin state dynamics in nine human cell types. Nature.

[77] Gerasimova A., Chavez L., Li B., Seumois G., Greenbaum J., Rao A. (2013). Predicting cell types and genetic variations contributing to disease by combining GWAS and epigenetic data. PLoS One.

[78] Rhie S.K., Coetzee S.G., Noushmehr H., Yan C., Kim J.M., Haiman C.A. (2013). Comprehensive functional annotation of seventy-one breast cancer risk Loci. PLoS One.

[79] Chen B., Dowlatshahi D., MacQueen G.M., Wang J.-F., Young L.T. (2001). Increased hippocampal bdnf immunoreactivity in subjects treated with antidepressant medication. Biol Psychiatry.

[80] Dwivedi Y., Rizavi H.S., Conley R.R., Roberts R.C., Tamminga C.A., Pandey G.N. (2003). Altered gene expression of brain-derived neurotrophic factor and receptor tyrosine kinase B in postmortem brain of suicide subjects. Arch Gen Psychiatry.

[81] Thompson Ray M., Weickert C.S., Wyatt E., Webster M.J. (2011). Decreased BDNF, trkB-TK + and GAD67 mRNA expression in the hippocampus of individuals with schizophrenia and mood disorders. J Psychiatry Neurosci.

[82] Ray M.T., Shannon Weickert C., Webster M.J. (2014). Decreased BDNF and TrkB mRNA expression in multiple cortical areas of patients with schizophrenia and mood disorders. Transl Psychiatry.

[83] Pruunsild P., Sepp M., Orav E., Koppel I., Timmusk T. (2011). Identification of cis-elements and transcription factors regulating neuronal activity-dependent transcription of human BDNF gene. J Neurosci.

[84] Lim Y.Y., Villemagne V.L., Laws S.M., Pietrzak R.H., Snyder P.J., Ames D. (2015). APOE and BDNF polymorphisms moderate amyloid beta-related cognitive decline in preclinical Alzheimer's disease. Mol Psychiatry.

[85] Zuccato C., Tartari M., Crotti A., Goffredo D., Valenza M., Conti L. (2003). Huntingtin interacts with REST/NRSF to modulate the transcription of NRSE-controlled neuronal genes. Nat Genet.

[86] Shimojo M., Hersh L.B. (2006). Characterization of the REST/NRSF-interacting LIM domain protein (RILP): localization and interaction with REST/NRSF. J Neurochem.

[87] Bassuk A.G., Wallace R.H., Buhr A., Buller A.R., Afawi Z., Shimojo M. (2008). A homozygous mutation in human PRICKLE1 causes an autosomal-recessive progressive myoclonus epilepsy–ataxia syndrome. Am J Hum Genet.

[88] Tzschach A., Lenzner S., Moser B., Reinhardt R., Chelly J., Fryns J.P. (2006). Novel JARID1C/SMCX mutations in patients with X-linked mental retardation. Hum Mutat.

[89] Guan J.S., Haggarty S.J., Giacometti E., Dannenberg J.H., Joseph N., Gao J. (2009). HDAC2 negatively regulates memory formation and synaptic plasticity. Nature.

[90] Graff J., Rei D., Guan J.S., Wang W.Y., Seo J., Hennig K.M. (2012). An epigenetic blockade of cognitive functions in the neurodegenerating brain. Nature.

[91] Abuhatzira L., Makedonski K., Kaufman Y., Razin A., Shemer R. (2007). MeCP2 deficiency in the brain decreases BDNF levels by REST/CoREST-mediated repression and increases TRKB production. Epigenetics.

[92] Paller K.A., Acharya A., Richardson B.C., Plaisant O., Shimamura A.P., Reed B.R. (1997). Functional neuroimaging of cortical dysfunction in alcoholic Korsakoff's syndrome. J Cogn Neurosci.

[93] Tateno M., Saito T. (2008). Biological studies on alcohol-induced neuronal damage. Psychiatry Investig.

